# Supplementary material for: Prevalence of Dry Eye Symptoms and Associated Risk Factors among University Students in Poland
Source: Int J Environ Res Public Health. 2023 Jan 11;20(2):1313. doi: 10.3390/ijerph20021313 (PMC9859544; doi:10.3390/ijerph20021313)
Supplement: Supplementary file 1 [file ijerph-20-01313-s001.zip › ijerph-2047275-supplementary.pdf]

1. Sex
a) Woman
b) Man

2. Age
a) <20 years
b) 20-25 years old
c) 26-30 years old
d) >30 years

3. Place of residence
a) City
b) Village

4. Field of study
a) Medical
b) Other than medical

5. Name of the field of study /Year of study
a) 1 year of first-cycle / uniform master's studies
b) 2nd year of first-cycle / long-cycle Master's studies
c) 3rd year of first-cycle / long-cycle Master's studies
d) 1 year of second-cycle studies / 4-year of uniform master's studies
e) 2nd year of second-cycle studies / 5th year of uniform master's studies
f) 3rd year of second-cycle studies / 6th year of uniform master's studies

6. Do you have a refractive error?

a) lack
b) myopia
c) hyperopia
d) astigmatism

7.Have you had surgery to correct your refractive error in the past?

a) YES
b) NO

8.Do you use contact lenses?

a) YES
b) NO

9.If you wear contact lenses, for how long?

a) year
b) 2-3 years
c) >3 years

10.If you wear contact lenses, do you tolerate them well?

a) YES
b) NO

11.Do you have any eye diseases (cataracts, glaucoma, other)?

a) YES
b) NO

12.Have you ever had eye injuries?

a) YES
b) NO

13.Do you have the following diseases?

a) hypertension
b) heart diseases
c) depression
d) other mental illnesses
e) thyroid disease
f) diabetes
g) allergy
h) rosacea
i) autoimmune diseases (RA, lupus, systemic sclerosis, AS, Sjogren's syndrome, others)
j) vitamin deficiency AND
k) neurological diseases (MS, neuropathies)
l) amyloidosis
m) sarcoidosis
n) hormonal disorders

14.Which of the following drugs are you taking on a regular basis?

a) β-blockers
b) diuretics
c) psychotrop medicines
d) antihistamines
e) painkillers
f) glucocorticoids (GCS)
g) anabolic steroids
h) oral contraceptives
i) other hormonal drugs

15.Do you use tobacco products on a regular, daily basis?

a) YES
b) NO

16.Do you have any other addictions? If so, please enter which ones.

17.Have you had a Covid-19 infection?

a) YES
b) NO

18.Have you been vaccinated against Covid-19?

a) Yes, full dose (two doses of a two-dose vaccine or one single-dose vaccine)
b) Yes, with a partial dose (one dose of a two-dose vaccine)
c) Yes, full dose and third booster
d) No

19.How would you rate your overall stress level?

a) 0 Lack of stress
b) 1
c) 2
d) 3
e) 4
f) 5 Maximum stress

Ocular Surface Disease Index (OSDI)

Have you experienced any of the following during the last week:

20.Eyes that are sensitive to light?

a) All of the time
b) Most of the time
c) Half of the time
d) Some of the time
e) None of the time

21.Eyes that feel gritty?

a) All of the time
b) Most of the time
c) Half of the time
d) Some of the time
e) None of the time

22.Painful or sore eyes?

a) All of the time
b) Most of the time
c) Half of the time
d) Some of the time
e) None of the time

23.Blurred vision?

a) All of the time
b) Most of the time

c) Half of the time
d) Some of the time
e) None of the time

24.Poor vision?

a) All of the time
b) Most of the time
c) Half of the time
d) Some of the time
e) None of the time

Have problems with your eyes limited you in performing any of the following during the last week:

25.Reading?

a) All of the time
b) Most of the time
c) Half of the time
d) Some of the time
e) None of the time

26.Driving at night?

a) All of the time
b) Most of the time
c) Half of the time
d) Some of the time
e) None of the time

27. Working with a computer or bank machine ?

a) All of the time
b) Most of the time
c) Half of the time
d) Some of the time
e) None of the time

28. Watching TV?

a) All of the time
b) Most of the time
c) Half of the time
d) Some of the time
e) None of the time

Have your eyes felt uncomfortable in any of the following situations during the last week:

29.Windy conditions?

a) All of the time
b) Most of the time

c) Half of the time
d) Some of the time
e) None of the time

30.Places or areas with low humidity (very dry)?

a) All of the time
b) Most of the time
c) Half of the time
d) Some of the time
e) None of the time

31. Areas that are air conditioned?

a) All of the time
b) Most of the time
c) Half of the time
d) Some of the time
e) None of the time

DEQ-5

32.During a typical day in the past month, how often did your eyes feel discomfort?

a) never
b) rarely
c) sometimes
d) frequently
e) constantly

33.When your eyes feel discomfort, how intense was this feeling of discomfort at the end of the day, within two hours of going to bed?

a) 0 - never have it
b) 1
c) 2
d) 3
e) 4
f) 5 - very intense

34.During a typical day in the past month, how often did your eyes feel dry?

a) never
b) rarely
c) sometimes
d) frequently
e) constantly

35.When your eyes felt dry, how intense was this feeling of dryness at the end of the day, within two hours of going to bed?

a) 0 - never have it
b) 1
c) 2
d) 3
e) 4
f) 5 - very intense

36.During a typical day in the past month, how often did your eyes look or feel excessively watery?

a) never
b) rarely
c) sometimes
d) frequently
e) constantly

37.How many hours a day do you spend in close proximity to the screen of an electronic device, e.g. mobile phone, computer, tablet, reader (not taking into account a TV set)?

a) <4
b) 4-8
c) >8

38.How many hours a day do you spend in front of the screen of an electronic device (not taking into account a TV set), without taking breaks?

a) <1
b) 1-2
c) 3-4
d) >4

39.How many hours a day do you spend outdoors?

a) <1
b) 1-4
c) >4

40.How many hours a day do you spend in air-conditioned interiors?

a) <1
b) 1-4
c) >4
